# Arylquin 1 (Potent Par-4 Secretagogue) Inhibits Tumor Progression and Induces Apoptosis in Colon Cancer Cells

**DOI:** 10.3390/ijms23105645

**Published:** 2022-05-18

**Authors:** Yi-Ting Chen, Tzu-Ting Tseng, Hung-Pei Tsai, Ming-Yii Huang

**Affiliations:** 1 Department of Pathology, Kaohsiung Medical University Hospital, Kaohsiung 80708, Taiwan; yt0728@gmail.com (Y.-T.C.); cawaii7992@gmail.com (T.-T.T.); 2 Department of Pathology, Faculty of Medicine, College of Medicine, Kaohsiung Medical University, Kaohsiung 80708, Taiwan; 3 Department of Surgery, Division of Neurosurgery, Kaohsiung Medical University Hospital, Kaohsiung 80708, Taiwan; carbugino@gmail.com; 4 Department of Radiation Oncology, Kaohsiung Medical University Hospital, Kaohsiung 80708, Taiwan; 5 Cancer Center, Kaohsiung Medical University Hospital, Kaohsiung 80708, Taiwan; 6 Department of Radiation Oncology, Faculty of Medicine, College of Medicine, Kaohsiung Medical University, Kaohsiung 80708, Taiwan

**Keywords:** Arylquin 1, colon cancer, tumor progression, apoptosis

## Abstract

Colorectal cancer (CRC) is one of the most common gastrointestinal cancers worldwide. Current therapeutic strategies mainly involve surgery and chemoradiotherapy; however, novel antitumor compounds are needed to avoid drug resistance in CRC, as well as the severe side effects of current treatments. In this study, we investigated the anticancer effects and underlying mechanisms of Arylquin 1 in CRC. The MTT assay was used to detect the viability of SW620 and HCT116 cancer cells treated with Arylquin 1 in a dose-dependent manner in vitro. Further, wound-healing and transwell migration assays were used to evaluate the migration and invasion abilities of cultured cells, and Annexin V was used to detect apoptotic cells. Additionally, Western blot was used to identify the expression levels of N-cadherin, caspase-3, cyclin D1, p-extracellular signal-regulated kinase (ERK), p-c-JUN N-terminal kinase (JNK), and phospho-p38, related to key signaling proteins, after administration of Arylquin 1. Xenograft experiments further confirmed the effects of Arylquin 1 on CRC cells in vivo. Arylquin 1 exhibited a dose-dependent reduction in cell viability in cultured CRC cells. It also inhibited cell proliferation, migration, and invasion, and induced apoptosis. Mechanistic analysis demonstrated that Arylquin 1 increased phosphorylation levels of ERK, JNK, and p38. In a mouse xenograft model, Arylquin 1 treatment diminished the growth of colon tumors after injection of cultured cancer cells. Arylquin 1 may have potential anticancer effects and translational significance in the treatment of CRC.

## 1. Introduction

Colorectal cancer (CRC) is the third most common malignancy in men and the second in women globally. In 2020, there were almost two million newly diagnosed cases of CRC, and it caused nearly one million deaths [1]. Wide excision for resectable cancer combined with postoperative chemotherapy remains the first choice of therapy in cases of CRC. Combined radiotherapy is now regarded as the standard treatment for advanced rectal cancer. However, not only chemotherapy but also radiation may cause unbearable side effects and toxicities to lead treatment failure due to early drug withdrawal. The development of innovative drugs that induce tumor apoptosis is the main focus of research into antineoplastic therapeutic agents. To find selective cancer-targeted therapeutics remains one of the greatest challenges.

Arylquin 1, a new molecule first identified by scientists at the University of Kentucky, is regarded as a secretagogue of prostate apoptosis response-4 (Par-4) in healthy cells. Par-4, regarded a tumor specific suppressor protein, is identified in normal cells, but it is often decreased or absent in neoplastic cells to evade the destiny of apoptosis [2]. Extracellular Par-4 can induce apoptosis through the binding of the 78 kDa glucose-regulated protein (GRP78) on tumor cell membranes [3]. Intracellularly, as seen in prostate cancers, Par-4 mainly activates the signaling pathway of the Fas death receptor and inhibits cellular pro-survival mechanisms [4]. Downregulation of Par-4 protein during tumorigenesis has been proposed associated with poor prognosis and treatment resistance in many solid tumors to highlight the importance as a critical event of therapeutic target agent. For example, Par-4 level was decreased in human renal cell carcinoma compared with normal renal tubular cells [5]. Low level of Par-4 was found correlated with low survival period in glioblastoma patients, and Tamoxifen-induced cell death was alleviated by Par-4 specific siRNA in vitro [6]. In breast cancer, knockdown of Par-4 increased the proliferation and reduced the chemosensitization [7]. However, gene therapy such as transfection with plasmids or adenoviral vectors is very complex and hundreds of challenges remain to be overcome. Therefore, replenishment of Par-4 by Arylquin 1 via sensitizing tumor cells to apoptosis is promising. Previously, Arylquin 1 has been reported to promote morphology changes and decrease viability in various cancer cells [8]; however, any anticancer effects of Arylquin 1 in CRC have yet to be determined.

The present study aimed to investigate the cytotoxic effects and mechanisms of Arylquin-1-induced cell death in CRC. To our knowledge, this is the first study to explore the therapeutic value of Arylquin 1 specifically in CRC.

## 2. Results

### 2.1. Arylquin-1-Attenuated Cell Viability in CRC Cell Lines

**Figure 1:**
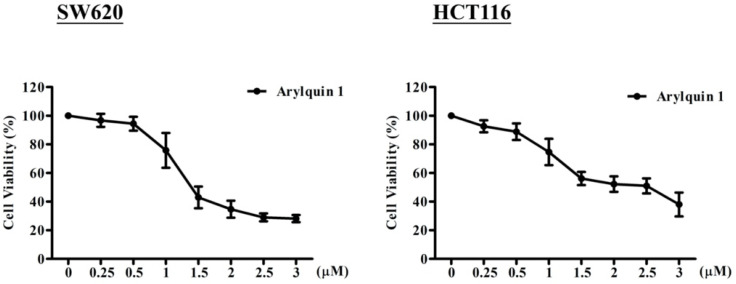
Viability of SW620 and HCT116 cells treated with different doses of Arylquin 1.

To explore the effects of Arylquin 1 on the proliferation of cultured CRC cells, cells were incubated with Arylquin 1 at different concentrations for 72 h, after which cell viability was assessed using the MTT assay. SW620 and HCT116 cells demonstrated reduced viability at doses of 0.25, 0.5, 1, 1.5, 2, 2.5, and 3 μM Arylquin 1 relative to the 0 μM control (Figure 1). These data showed a dose-dependent reduction in proliferation after Arylquin 1 treatment. The IC_50_ concentrations of Arylquin 1 were 1.8 and 2.3 μM for SW620 and HCT116 cells, respectively.

### 2.2. Arylquin-1-Inhibited Cell Migration, Invasion, and Epithelial–Mesenchymal Transition (EMT) in CRC Cells

**Figure 2:**
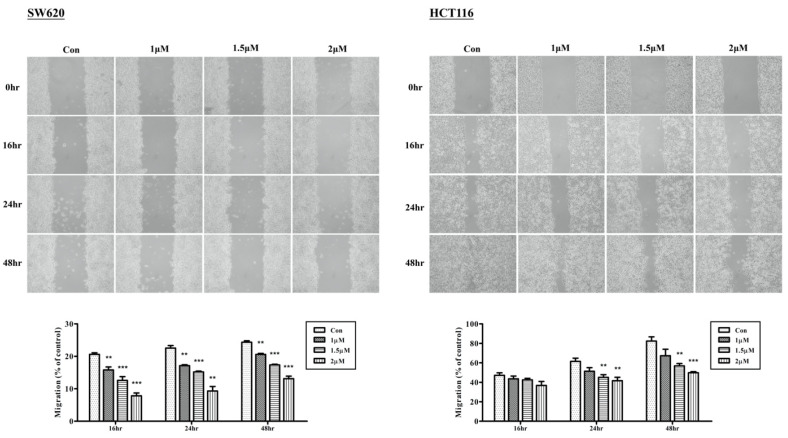
Wound-healing assay and percentage of cell migration in cultured SW620 and HCT116 cells 0, 16, 24, and 48 h after treatment with Arylquin 1; quantitative data are expressed as mean ± SEM. ** *p* < 0.01 and *** *p* < 0.001 compared to the control group.

To investigate the effects of cell migration after treatment with Arylquin 1, we used a wound-healing assay and compared the results between a control group and the Arylquin-1-treated groups. In both SW620 and HCT116 cells, 1, 1.5, and 2 μM Arylquin 1 markedly inhibited cell migration at 16, 24, and 48 h after administration (Figure 2). These data also indicated the dose-dependent effects of Arylquin 1 on cell migration in CRC cells.

**Figure 3:**
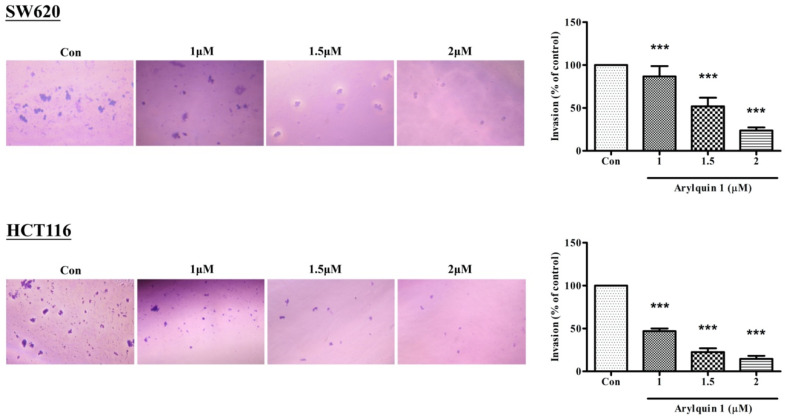
Wound-healing assay and percentage of cell migration in cultured SW620 and HCT116 cells 24 h after treatment with Arylquin 1; quantitative data are expressed as mean ± SEM. *** *p* < 0.001 compared to the control group.

To evaluate cell invasion, we used a Matrigel invasion assay. In both SW620 and HCT116 cells, 1, 1.5, and 2 μM Arylquin 1 significantly inhibited cell invasion 24 h after administration (Figure 3). As in the wound-healing and proliferation assays, Arylquin 1 inhibited the cell migration in CRC cells in a dose-dependent manner.

**Figure 4:**
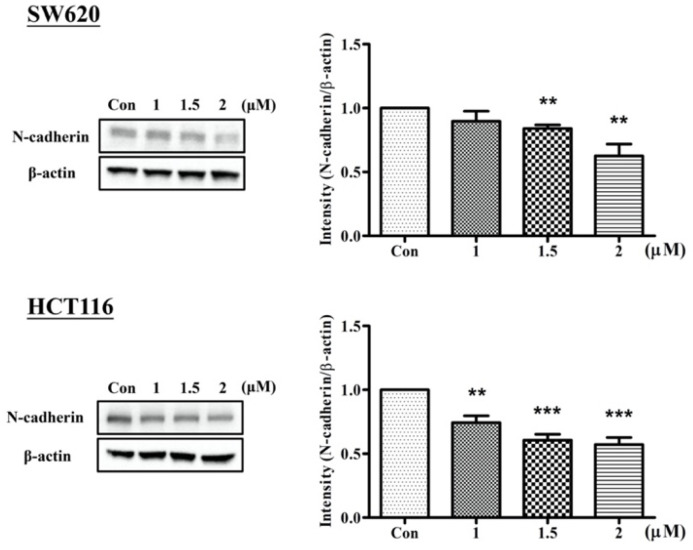
Western blot for N-cadherin, with β-actin-loading control, in SW620 and HCT116 cells treated with Arylquin 1; quantitative data are expressed as mean ± SEM; ** *p* < 0.01 and *** *p* < 0.001 compared to the control group.

Cadherins are markers of metastasis used to evaluate the ability of cells to undergo an epithelial–mesenchymal transition (EMT). SW620 and HCT116 cells treated with Arylquin 1 were subjected to Western blot (Figure 4), showing that N-cadherin levels were significantly lower in Arylquin-1-treated CRC cells in a dose-dependent manner, indicating that Arylquin 1 attenuates EMT in CRC.

### 2.3. Arylquin 1 Promotes Apoptosis in Cultured CRC Cells

**Figure 5:**
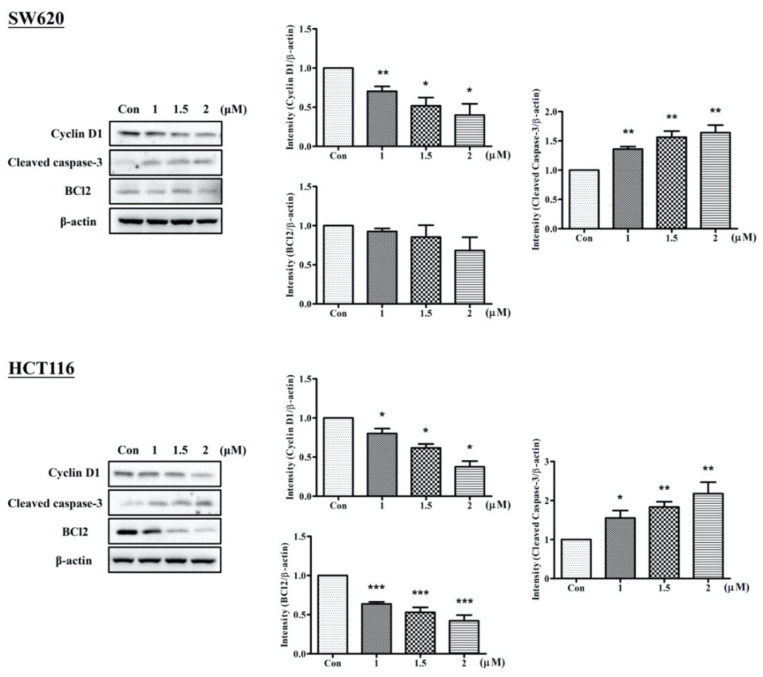
Western blot for Cyclin D1, cleaved caspase-3, and BCl2, with a β-actin-loading control, in SW620 and HCT116 cells 72 h after treated with Arylquin 1; quantitative data are expressed as mean ± SEM. * *p* < 0.05, ** *p* < 0.01, and *** *p* < 0.001 compared to the control group.

As shown in Figure 5, the highest level of cleaved caspase-3 and the lowest level of Cyclin-D1 were detected 72 h after treatment with a dose of 2 μM Arylquin 1, indicating that apoptotic activity is positively associated with Arylquin 1 dose strength in both SW620 and HCT116 cells. Moreover, BCl2 levels showed a tendency to significantly decrease after treatment with Arylquin 1 in HCT116 cells.

**Figure 6:**
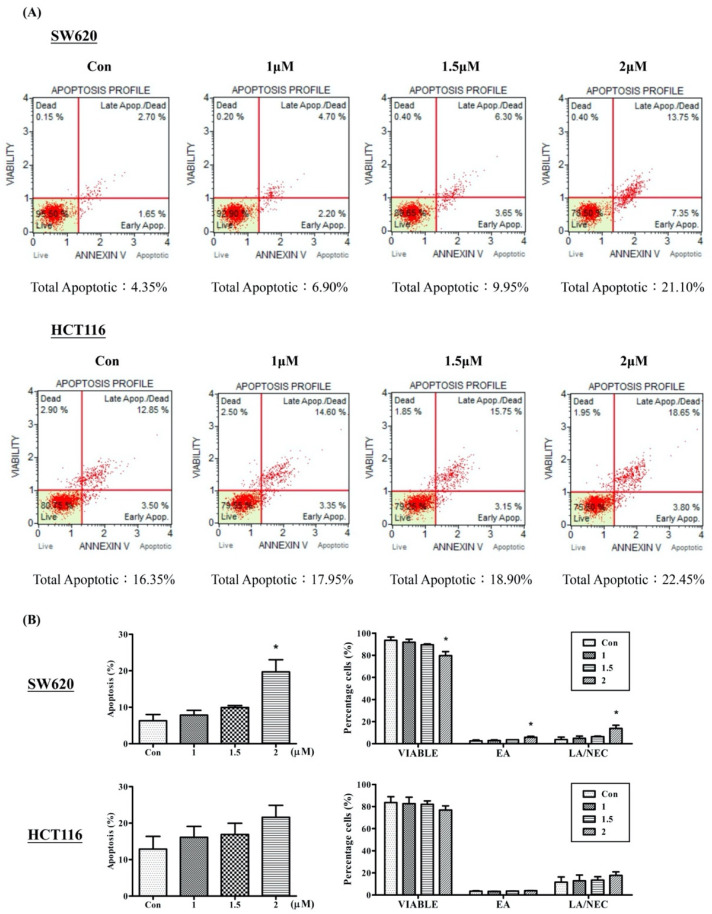
Annexin V/FITC Assay: (**A**) dot plot of Annexin V/FITC in SW620 and HCT116 cells after treatment with Arylquin 1; (**B**) quantification of Annexin V/FITC in SW620 and HCT116 cells. EA—early apoptosis; LA/NEC—late apoptosis/necrosis. All data are expressed as mean ± SEM. * *p* < 0.05 compared with corresponding controls.

The Annexin V/FITC assay was performed to identify apoptotic cells. A high proportion of viable cells was observed in the untreated control group in both SW620 (93.52 ± 3.1%) and HCT116 (83.65 ± 5.37%) cells (Figure 6). In both cell lines, a pattern of the cell population shifting from viable cells to early apoptotic stages to late apoptotic stages was observed, and this was associated with the Arylquin 1 dosage. However, a significant increase in early and late apoptotic/necrotic cells was observed after treatment with 2 μM Arylquin 1 in SW620 cells (19.68 ± 5%). These results revealed that Arylquin 1 can induce apoptosis and necrosis in a dose-dependent manner.

### 2.4. Arylquin 1 Regulated Apoptosis via the MAPK Pathway in CRC Cells

**Figure 7:**
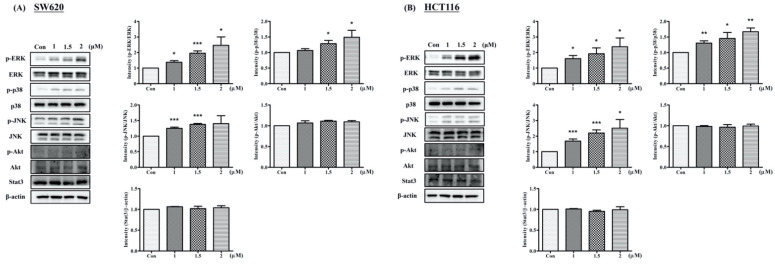
Western blot for p-ERK, ERK, p-p38, p38, p-JNK, JNK, p-Akt, Akt, and Stat3, with a β-actin-loading control, in (**A**) SW620 and (**B**) HCT116 cells treated with Arylquin 1; quantitative data are expressed as mean ± SEM; * *p* < 0.05, ** *p* < 0.01, and *** *p* < 0.001 compared to the control group.

To further clarify the signaling pathways involved and whether CRC cell viability decreased as a consequence of Arylquin-1-induced apoptosis, SW620 and HCT116 cells treated with Arylquin 1 were subjected to Western blot for proteins downstream of the mitogen-activated protein kinase (MAPK), Akt, and signal transducer and activator of transcription 3 (STAT3) pathways. The MAPK family consists of three major subfamilies of related proteins (extracellular-signal-regulated kinases [ERKs], c-Jun N-terminal kinase [JNK], and p38). In both SW620 and HCT116 cells, Arylquin 1 administration led to the upregulation of ERK, p38, and JNK expression (Figure 7).

### 2.5. Arylquin 1 Suppressed the Growth of CRC Cells in Mice

**Figure 8:**
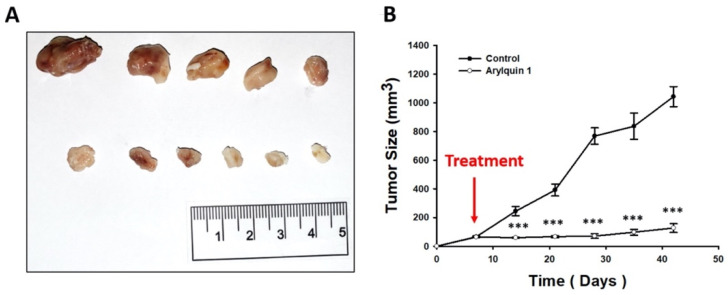
(**A**) Gross examination and (**B**) tumor growth curves of mouse xenografts of human colorectal cancer with and without Arylquin 1 treatment; quantitative data are expressed as mean ± SEM; *** *p* < 0.001 compared to the control group.

Having validated our cell model in vitro, we next evaluated the in vivo effects of Arylquin 1 on HCT116 tumor xenograft growth. Tumor or control (empty) cell suspensions were injected subcutaneously into the flanks of 6-week-old mice, and tumor growth was evaluated and registered periodically (Figure 8). Arylquin 1 was injected intraperitoneally on day 7 after cell implantation. Our results demonstrate a significant and marked reduction in tumor volume in the Arylquin 1 treatment group, from day 14 after cell injection until the end of the experiment at day 42 (all *p* < 0.01). The Arylquin 1 treatment group showed significantly slower tumor growth compared with the untreated control group, displaying final tumor volumes of 127 ± 69 and 1042 ± 157 mm^3^, respectively.

## 3. Discussion

CRC is one of the most commonly diagnosed cancers and is a leading cause of cancer-related death. Currently, the chemotherapeutic strategy for the treatment of advanced CRC is based on a variety of reagents, sometimes accompanied by lethal and dose-dependent adverse side effects. Thus, it is worth exploring potential drug substitutions to increase treatment effectiveness and cause fewer severe side effects in patients with CRC.

In the present study, various assays were used to investigate the antitumor effects of Arylquin 1 in human CRC. We first identified that treatment with Arylquin 1 inhibited growth in cultured SW620 and HCT116 cells with an IC_50_ of 1.8 and 2.3 μM, respectively. In mice injected with cultured human CRC cells, treatment with Arylquin 1 decreased tumor size. Arylquin 1 was also found to inhibit cell migration and invasion. Western blot showed that Arylquin 1 treatment led to downregulated N-cadherin expression.

Arylquin 1, a molecular compound regarded as a Par-4 secretagogue in healthy cells, was identified in 2014 by Rangnekar et al. [9]. This structure–activity study has been confirmed, with results showing that Arylquin 1 targets vimentin and causes the release of Par-4 [10]. Par-4, a 38 kDa tumor suppressor protein, is expressed in healthy cells, but it is usually downregulated or silenced in cancer cells to elude the induction of apoptosis [2]. Recently, the intracellular apoptotic effects of Par-4 and the ability of cancer cells to inhibit Par-4 release were carefully investigated [4]. Apoptosis induced by interactions between Par-4 and the tumor cell surface receptor GRP78 leads to programmed cell death [3,10]. Moreover, Min et al. reported that Arylquin 1 can induce lysosomal membrane permeabilization, with useful antitumor effects [8]. Chloroquine, another secretagogue of Par-4, has also been identified to induce apoptosis and inhibit tumor metastasis. Arylquin 1 and Chloroquine share a common pharmacophore [11]. Therefore, the potential cytotoxic activity of Arylquin 1 to bolster the release of Par-4 is a promising therapeutic advance.

In our study, the ability of inhibiting cell migration and invasion by Arylquin 1 treatment was noted. We found that Arylquin 1 attenuated N-cadherin expression in CRC. EMT has been identified to play an important role in tumor progression and metastasis. Upregulation of vimentin and N-cadherin regulated by a complex signaling pathway is the hallmark of EMT [12]. In previous studies, vimentin, which is highly involved in EMT and metastasis, was reported as the primary target of Arylquin 1 to inhibit the spread of lung cancer [9,13]. Silencing endogenous Par-4 using siRNA has also been shown to promote tumor growth and metastasis resulted in increased N-cadherin expression in pancreatic cancers [14]. Upregulation of Vimentin and N-cadherin were also detected associated with the interaction of Par-4 in cervical cancer during transforming growth factor (TGF)-β-induced EMT [15]. Furthermore, overexpression of Par-4 was proved to reduce EMT which was later promote apoptosis in pancreatic cancer [16]. It seems that our results may provide evidence that Aryqulin-1 may enhance the anti-EMT effects via Par-4 in tumor cells.

From the Annexin V/FITC assay in the present study, it was observed that both SW620 and HCT116 cells underwent apoptosis after Arylquin 1 treatment. The proportion of cells observed undergoing apoptosis increased as the dose increased. This shows that Arylquin 1 can induce apoptosis in a dose-dependent manner in both SW620 and HCT116 cells. It seems that CRC cell viability decreased is a consequence of Arylquin-1-induced apoptosis. There are two main pathways involved in apoptosis, the mitochondria-related intrinsic pathway and the death-receptor-related extrinsic pathway [17,18]. In the present study, downregulation of BCl2 and upregulation of caspase-3, which is involved in both intrinsic and extrinsic apoptosis pathways, were identified in HCT116 cells treated by Arylquin 1. Rangnekar et al. have previously shown that Par-4 upregulation induces apoptotic death in prostate cancer [19]. Wang et al. further reported a direct correlation between Par-4 levels and apoptotic activity induced by 5-fluorouracil in CRC [20]. Moreover, CRC tumor growth—namely the tumor growth rate as reflected by size after 42 days—was also suppressed by Arylquin 1 in mice in the present study, confirming the apoptosis-inducing effects of Arylquin 1.

Higher apoptosis percentage with a significant decrease in BCl2 expression was identified in HCT116 cells than SW620 cells in our data. As we know, the programmed cell death leaded by the interactions between Par-4 and the tumor cell surface receptor GRP78 has been investigated [2]. GRP78 is regarded as the central regulator of endoplasmic reticulum stress in apoptosis. Upregulation of GRP78 in tumor cells has been identified associated with reduce tumorigenicity and increase sensitivity to DNA crosslinking agents due to promote its localization in cell surface [21,22]. The oncogenic role of GRP78 draws attention to its value as a prognostic and drug response marker to mediate the therapeutic efficiency [23]. Therefore, the variable surface and cellular location of GRP78 expressions may alter and result in the different behaviors of SW620 and HCT116 cells.

In the present study, we found that both promoted apoptosis and increased expression of phosphorylated ERK in CRC treated with Arylquin 1 were observed, in addition to JNK and p38 upregulation. The MAPK signaling pathway, including ERK1/2, JNK/SAPK, and p38, is involved in cell proliferation, apoptosis, and metastasis of cancer cells, depending on the cell type and stimulus [24]. Therefore, modulating this pathway can provide a potential approach to treating CRC. ERK has been reported to promote cell growth and control migration [25,26]. On the other hand, tumor-necrosis-factor-associated apoptosis-inducing ligand and TGF-β were identified to induce apoptosis via ERK-mediated upregulation of death receptors in colon cancer cells [27,28]. Activation of JNK has also been shown to be involved in cell survival, proliferation, migration, invasion, and cell death [29,30]. Previously, ERK and JNK were reported to participate in the regulation of apoptosis (intrinsic and extrinsic; [30,31]). JNK can activate apoptotic pathway by regulating the activities of mitochondria directly or upregulating proapoptotic genes through transcriptional factors [32]. Activation of JNK pathway of apoptosis induced by Vernodalin or TRAIL was found in colon cancer cells [33,34]. p38 is also involved in the signal integration of migration, metastasis, and apoptosis in cancer [35]. In HeLa cells, Lee et al. reported that TRAIL induced apoptosis via p38 activation stimulated by reactive oxygen species [36]. Our results support the crucial role of MAPK in the regulation of proliferation, migration, metastasis, and apoptotic processes in CRC cells. Since these proteins act on tumor behavior, their dysregulation by Arylquin 1 highlights the novel mechanisms that may produce its antiproliferative, anti-invasive, and apoptotic functions.

## 4. Materials and Methods

### 4.1. Cell Culture

SW620 and HCT116 cells were purchased from the Bioresource Collection and Research Center (Taiwan) and cultured in Dulbecco’s Modified Eagle Medium (DMEM) (Gibco; 12800-017, Waltham, MA; USA) with 10% fetal bovine serum (FBS) at 37 °C in an atmosphere of 5% CO_2_.

### 4.2. Cell Viability

Cell lines were seeded on a 24-well plate at a density of about 3 × 10^4^ cells in 500 μL of DMEM with 10% FBS in each well. The viable cell counts were performed following the MTT assay after culturing with different doses of Arylquin 1 (0, 0.25, 0.5, 1, 1.5, 2, 2.5, and 3 μM) for 72 h to identify the IC_50_ dose.

### 4.3. Migration and Invasion Assays In Vitro

Cell migration was investigated using the wound-healing assay (Cat. Nr. 80209; Ibidi GmbH, Graefelfing, Germany) using two-sided wound gaps. The wound-healing assay was performed in 24-well plates seeded with 3 × 10^5^ cells per insert and cultured at 37 °C for 24 h, after which 0, 1, 1.5, or 2 μM Arylquin 1 was added to the wells. After 24 h, these cells were washed twice with phosphate-buffered saline (PBS) and photographed at 16, 24, and 48 h after washing. The transwell migration assay (COR3452; Corning Inc., Corning, NY, USA) was used to measure cell invasion in vitro. Incubated cells treated with 0, 1, 1.5, or 2 μM Arylquin 1 were seeded at 5 × 10^3^ cells per insert, and the lower chamber of the transwell assay was filled with 0.5 mL DMEM with 10% FBS. After 24 h, cells that remained on the upper surface of the transwell membrane were removed by a cotton swab. Cells that had passed through the transwell membrane to the bottom of the insert were fixed using formalin, stained using 0.5% crystal violet, and quantified via manual counts from photographs of six randomly selected fields.

### 4.4. Protein Extraction and Western Blot

All samples were prepared in 100 μL of RIPA lysis buffer, and 30 μg protein from each sample was loaded in the wells of a sodium dodecyl sulfate–polyacrylamide gel electrophoresis gel; gels were run at 80 V for 2 h. The separated proteins were then transferred from the gel to a polyvinyl difluoride membrane with a 400 mA current for 2 h. After 1 h in blocking buffer, the membranes were incubated with primary antibodies overnight, followed by incubation with secondary antibodies for 1 h. The primary antibodies used were anti-β-actin (MAB1501R; Sigma-Aldrich; Burlington, MA, USA), anti-p-p38 (9211S; Cell Signaling; Danvers, MA, USA), anti-p38 (9212S; Cell Signaling; Danvers, MA, USA), anti-p-JNK (4668S; Cell Signaling; Danvers, MA, USA), anti-JNK (9252S; Cell Signaling; Danvers, MA, USA), anti-p-ERK (9101S; Cell Signaling; Danvers, MA, USA), anti-ERK (9102S; Cell Signaling; Danvers, MA, USA), anti-p-Akt (9271S; Cell Signaling; Danvers, MA, USA), anti-Akt (9272S; Cell Signaling; Danvers, MA, USA), anti-Stat3 (sc-8019; Santa Cruz, CA, USA), anti-N-cadherin (22018-1-AP; Proteintech; Rosemont, IL, USA), anti-Cyclin D1 (60186-1-Ig; Proteintech; Rosemont, IL, USA), anti-cleaved caspase-3 (9661S; Cell Signaling; Danvers, MA, USA), and anti-BCl2 (4223S; Cell Signaling; Danvers, MA, USA). Horseradish peroxidase (HRP)-conjugated secondary antibodies were goat anti-rabbit IgG (H + L)-HRP (C04003; Croyez, Taipei, Taiwan) and goat anti-mouse IgG (H + L)-HRP (C04001; Croyez, Taipei, Taiwan). Signals were detected using an enhanced chemiluminescent solution (Western Lightning Plus; PerkinElmer, Waltham, MA, USA) with a chemiluminescence imager (Minichemi; Thermo Fisher Scientific, Waltham, MA, USA).

### 4.5. Flow Cytometry

Cells were transfected to 6-well plates and treated with 0, 1, 1.5, or 2 μM Arylquin 1 for 72 h. Both detached and attached cells were centrifuged at 1000 rpm for 5 min, washed once with 1× PBS, then treated according to the manufacturer’s instructions using the Muse^®^ Annexin V and Dead Cell Kit (Cat. No. MCH100105; MilliporeSigma, Burlington, MA, USA).

### 4.6. Animal Model

Six-week-old male NU/NU nude mice were obtained from BioLasco Taiwan (Taipei, Taiwan). All animal experiments followed the protocols of the Institutional Animal Care and Use Committee of Kaohsiung Medical University (IACUC Approval No: 110266) and were performed according to the Guiding Principles for the Care and Use of Laboratory Animals. Mice were acclimatized to a 12:12 h light/dark cycle at 24 ± 1 °C with ad libitum access to food and water. Human HCT116 cells were chosen for tumor xenograft to evaluate the effects of Arylquin 1 on tumor growth, invasion, and metastasis of colon cancer according to previous studies [37,38,39]. HCT116 cells were subcutaneously injected at a density of 1 × 10^7^ in NU/NU mice. One week after injection, 150 uM/kg Arylquin 1 was injected intraperitoneally. Tumor volume (mm^3^) was measured three times a week, calculated as (length × width^2^)/2 on days 7, 10, 14, 21, 28, 35, and 42. Mice were killed 42 days after the injection of tumor cells.

### 4.7. Statistical Analysis

All statistical analyses were performed with SPSS 19.0 (IBM Corp., Armonk, NY, USA). Intensities of Western blot bands were digitally analyzed using ImageJ software. All tests were two-sided, and a *p*-value less than 0.05 was considered statistically significant.

## 5. Conclusions

In summary, the present study demonstrates for the first time that Arylquin 1, as a secretagogue of Par-4, can inhibit the proliferation of CRC cells in vitro and can decrease migration, invasion, and metastasis. Moreover, promoting apoptosis in vitro and suppressing tumor size in vivo were also observed. In conclusion, Arylquin 1 may be used as a potential anticancer drug with strong translational significance in the treatment of CRC.

## Data Availability

The datasets used and/or analyzed during the current study are available from the corresponding author on reasonable request.

## References

[1] FIAfRoC L. Global Cancer Observatory: Cancer Today. https://gcoiarcfr/today/home.

[2] Hebbar N., Wang C., Rangnekar V.M. (2012). Mechanisms of apoptosis by the tumor suppressor Par-4. J. Cell Physiol..

[3] Burikhanov R., Zhao Y., Goswami A., Qiu S., Schwarze S.R., Rangnekar V.M. (2009). The tumor suppressor Par-4 activates an extrinsic pathway for apoptosis. Cell.

[4] Chakraborty M., Qiu S.G., Vasudevan K.M., Rangnekar V.M. (2001). Par-4 drives trafficking and activation of Fas and Fasl to induce prostate cancer cell apoptosis and tumor regression. Cancer Res..

[5] Cook J., Krishnan S., Ananth S., Sells S.F., Shi Y., Walther M.M., Linehan W.M., Sukhatme V.P., Weinstein M.H., Rangnekar V.M. (1999). Decreased expression of the pro-apoptotic protein Par-4 in renal cell carcinoma. Oncogene.

[6] Jagtap J.C., Parveen D., Shah R.D., Desai A., Bhosale D., Chugh A., Ranade D., Karnik S., Khedkar B., Mathur A. (2015). Secretory prostate apoptosis response (Par)-4 sensitizes multicellular spheroids (MCS) of glioblastoma multiforme cells to tamoxifen-induced cell death. FEBS Open Bio.

[7] Pereira M.C., de Bessa-Garcia S.A., Burikhanov R., Pavanelli A.C., Antunes L., Rangnekar V.M., Nagai M.A. (2013). Prostate apoptosis response-4 is involved in the apoptosis response to docetaxel in MCF-7 breast cancer cells. Int. J. Oncol..

[8] Min K.J., Shahriyar S.A., Kwon T.K. (2020). Arylquin 1, a potent Par-4 secretagogue, induces lysosomal membrane permeabilization-mediated non-apoptotic cell death in cancer cells. Toxicol. Res..

[9] Burikhanov R., Sviripa V.M., Hebbar N., Zhang W., Layton W.J., Hamza A., Zhan C.G., Watt D.S., Liu C., Rangnekar V.M. (2014). Arylquins target vimentin to trigger Par-4 secretion for tumor cell apoptosis. Nat. Chem. Biol..

[10] Sviripa V.M., Burikhanov R., Obiero J.M., Yuan Y., Nickell J.R., Dwoskin L.P., Zhan C.G., Liu C., Tsodikov O.V., Rangnekar V.M. (2016). Par-4 secretion: Stoichiometry of 3-arylquinoline binding to vimentin. Org. Biomol. Chem..

[11] Burikhanov R., Hebbar N., Noothi S.K., Shukla N., Sledziona J., Araujo N., Kudrimoti M., Wang Q.J., Watt D.S., Welch D.R. (2017). Chloroquine-Inducible Par-4 Secretion Is Essential for Tumor Cell Apoptosis and Inhibition of Metastasis. Cell Rep..

[12] Kang Y., Massagué J. (2004). Epithelial-mesenchymal transitions: Twist in development and metastasis. Cell.

[13] Usman S., Waseem N.H., Nguyen T.K.N., Mohsin S., Jamal A., Teh M.T., Waseem A. (2021). Vimentin Is at the Heart of Epithelial Mesenchymal Transition (EMT) Mediated Metastasis. Cancers.

[14] Katoch A., Jamwal V.L., Faheem M.M., Kumar S., Senapati S., Yadav G., Gandhi S.G., Goswami A. (2021). Overlapping targets exist between the Par-4 and miR-200c axis which regulate EMT and proliferation of pancreatic cancer cells. Transl. Oncol..

[15] Chaudhry P., Fabi F., Singh M., Parent S., Leblanc V., Asselin E. (2014). Prostate apoptosis response-4 mediates TGF-beta-induced epithelial-to-mesenchymal transition. Cell Death Dis..

[16] Mohd Faheem M., Rasool R.U., Ahmad S.M., Jamwal V.L., Chakraborty S., Katoch A., Gandhi S.G., Bhagat M., Goswami A. (2020). Par-4 mediated Smad4 induction in PDAC cells restores canonical TGF-β/Smad4 axis driving the cells towards lethal EMT. Eur. J. Cell Biol..

[17] Obeng E. (2021). Apoptosis (programmed cell death) and its signals—A review. Braz. J. Biol..

[18] Elmore S. (2007). Apoptosis: A review of programmed cell death. Toxicol. Pathol..

[19] Sells S.F., Wood D.P., Joshi-Barve S.S., Muthukumar S., Jacob R.J., Crist S.A., Humphreys S., Rangnekar V.M. (1994). Commonality of the gene programs induced by effectors of apoptosis in androgen-dependent and -independent prostate cells. Cell Growth Differ..

[20] Wang B.D., Kline C.L., Pastor D.M., Olson T.L., Frank B., Luu T., Sharma A.K., Robertson G., Weirauch M.T., Patierno S.R. (2010). Prostate apoptosis response protein 4 sensitizes human colon cancer cells to chemotherapeutic 5-FU through mediation of an NF kappaB and microRNA network. Mol. Cancer.

[21] Hardy B., Raiter A., Yakimov M., Vilkin A., Niv Y. (2012). Colon cancer cells expressing cell surface GRP78 as a marker for reduced tumorigenicity. Cell Oncol..

[22] Belfi C.A., Chatterjee S., Gosky D.M., Berger S.J., Berger N.A. (1999). Increased sensitivity of human colon cancer cells to DNA cross-linking agents after GRP78 up-regulation. Biochem. Biophys. Res. Commun..

[23] Gifford J.B., Hill R. (2018). GRP78 Influences Chemoresistance and Prognosis in Cancer. Curr. Drug Targets.

[24] Burotto M., Chiou V.L., Lee J.M., Kohn E.C. (2014). The MAPK pathway across different malignancies: A new perspective. Cancer.

[25] Park J.I. (2014). Growth arrest signaling of the Raf/MEK/ERK pathway in cancer. Front. Biol..

[26] Wu W.S., Wu J.R., Hu C.T. (2008). Signal cross talks for sustained MAPK activation and cell migration: The potential role of reactive oxygen species. Cancer Metastasis Rev..

[27] Kim B., Seo J.H., Lee K.Y., Park B. (2020). Icariin sensitizes human colon cancer cells to TRAIL-induced apoptosis via ERK-mediated upregulation of death receptors. Int. J. Oncol..

[28] Zhao Y., Xia S., Cao C., Du X. (2019). Effect of TGF-β1 on Apoptosis of Colon Cancer Cells Via the ERK Signaling Pathway. J. BUON.

[29] Tournier C. (2013). The 2 Faces of JNK Signaling in Cancer. Genes Cancer.

[30] Dhanasekaran D.N., Reddy E.P. (2017). JNK-signaling: A multiplexing hub in programmed cell death. Genes Cancer.

[31] Cagnol S., Chambard J.C. (2010). ERK and cell death: Mechanisms of ERK-induced cell death—Apoptosis, autophagy and senescence. FEBS J..

[32] Dhanasekaran D.N., Reddy E.P. (2008). JNK signaling in apoptosis. Oncogene.

[33] Mohebali N., Pandurangan A.K., Mustafa M.R., Anandasadagopan S.K., Alagumuthu T. (2020). Vernodalin induces apoptosis through the activation of ROS/JNK pathway in human colon cancer cells. J. Biochem. Mol. Toxicol..

[34] Guo X., Meng Y., Sheng X., Guan Y., Zhang F., Han Z., Kang Y., Tai G., Zhou Y., Cheng H. (2017). Tunicamycin enhances human colon cancer cells to TRAIL-induced apoptosis by JNK-CHOP-mediated DR5 upregulation and the inhibition of the EGFR pathway. Anticancer Drugs.

[35] Wagner E.F., Nebreda A.R. (2009). Signal integration by JNK and p38 MAPK pathways in cancer development. Nat. Rev. Cancer.

[36] Lee M.W., Park S.C., Yang Y.G., Yim S.O., Chae H.S., Bach J.H., Lee H.J., Kim K.Y., Lee W.B., Kim S.S. (2002). The involvement of reactive oxygen species (ROS) and p38 mitogen-activated protein (MAP) kinase in TRAIL/Apo2L-induced apoptosis. FEBS Lett..

[37] Yang M., Li W.Y., Xie J., Wang Z.L., Wen Y.L., Zhao C.C., Tao L., Li L.F., Tian Y., Sheng J. (2021). Astragalin Inhibits the Proliferation and Migration of Human Colon Cancer HCT116 Cells by Regulating the NF-κB Signaling Pathway. Front. Pharmacol..

[38] Rajput A., San Martin I.D., Rose R., Beko A., Levea C., Sharratt E., Mazurchuk R., Hoffman R.M., Brattain M.G., Wang J. (2008). Characterization of HCT116 human colon cancer cells in an orthotopic model. J. Surg. Res..

[39] Phang C.W., Abd Malek S.N., Karsani S.A. (2021). Flavokawain C exhibits antitumor effects on in vivo HCT 116 xenograft and identification of its apoptosis-linked serum biomarkers via proteomic analysis. Biomed. Pharmacother..

